# Molecular Research in Cardiovascular Disease, 3rd Edition

**DOI:** 10.3390/ijms27135844

**Published:** 2026-06-29

**Authors:** Maria Dorobantu

**Affiliations:** Department of Medical Sciences, Romanian Academy, 010071 Bucharest, Romania; maria.dorobantu@gmail.com

Cardiovascular medicine has entered a paradoxical era. Never before have we possessed such sophisticated diagnostic tools, advanced imaging modalities, powerful computational methods, and effective therapeutic options. Yet cardiovascular disease remains the leading cause of death worldwide. The explanation may lie in a realization that has become increasingly evident over the past decade: cardiovascular disease is rarely the consequence of a single pathological process. Rather, it emerges from the interaction of multiple biological systems operating across molecular, cellular, tissue, and organ levels.

The studies assembled in the third edition of *Molecular Research in Cardiovascular Disease* reflect this changing perspective. Although they investigate seemingly distinct conditions—including coronary artery disease, heart failure, thoracoabdominal aortic aneurysms, cardiomyopathies, vascular dysfunction, and chemotherapy-induced cardiac injury—they collectively reveal a field moving away from reductionist views and toward a more integrated understanding of cardiovascular biology.

One of the strongest themes emerging from this Special Issue is the central role of inflammation as a common denominator across cardiovascular disorders. Inflammation is no longer viewed merely as a consequence of disease but increasingly as an active driver of disease initiation, progression, and clinical instability. The association between inflammatory biomarkers and coronary lesion complexity reported by Popa-Fotea et al. illustrates how systemic immune activation is directly linked to plaque vulnerability and residual cardiovascular risk (Contribution 1). At the vascular level, the review by Niculescu et al. further expands this concept by portraying perivascular adipose tissue as an active immunometabolic organ capable of shaping local vascular biology through continuous molecular crosstalk (Contribution 2). This interpretation is consistent with recent mechanistic and translational literature describing atherosclerosis as a lipid-driven inflammatory disease in which macrophage activation, immune-cell heterogeneity, defective resolution, and residual inflammatory risk create plausible links between systemic biomarkers and plaque vulnerability [1,2,3].

Equally prominent throughout this collection is the growing recognition that metabolism is inseparable from cardiovascular function. The heart has traditionally been viewed as a mechanical pump, yet it is equally an energy-intensive metabolic organ. The review by Pietrangelo et al. highlights how mitochondrial dysfunction, altered bioenergetics, and oxidative stress contribute to the pathogenesis of a broad spectrum of cardiovascular diseases (Contribution 3). At the cellular level, the work of Líšková et al. demonstrates how modulation of oxidative stress and Wnt/β-catenin signaling can influence susceptibility to doxorubicin-induced cardiotoxicity, emphasizing the intricate relationship between cellular metabolism and cardiac resilience (Contribution 4). Recent syntheses further support mitochondrial dysfunction as a nodal mechanism connecting impaired bioenergetics, oxidative stress, calcium handling, and inflammatory signaling, thereby reinforcing the relevance of metabolic remodeling across ischemic, hypertensive, and heart failure phenotypes [4].

Another recurring concept is extracellular matrix remodeling. Once considered a largely structural process, matrix turnover is now recognized as a highly dynamic biological mechanism governing tissue adaptation, repair, and disease progression. This perspective is evident in several contributions within the Special Issue. Doukas et al. identify plasma desmosine as a marker of ongoing elastin degradation in thoracoabdominal aortic aneurysms, linking circulating biomarkers to active proteolytic remodeling of the vascular wall (contribution 6). Similarly, Ceraolo et al. demonstrate that targeted CAR-T cell therapy can modulate fibrosis and extracellular matrix remodeling in Duchenne muscular dystrophy-associated cardiomyopathy, illustrating how molecular interventions may directly influence tissue architecture and function (Contribution 5). Contemporary experimental and translational studies similarly show that matrix biology is not a passive consequence of injury: post-ischemic models reveal coordinated transcriptomic, proteomic, glycomic, inflammatory, and fibrotic remodeling, while broader cardiovascular literature emphasizes the extracellular matrix as an active regulator of cellular behavior, repair, and maladaptive fibrosis [5,6].

Perhaps most importantly, the contributions published in this Special Issue reflect the ongoing transition from descriptive cardiovascular medicine toward precision cardiovascular medicine. Biomarkers are increasingly expected to do more than merely confirm diagnosis; they are now being developed to characterize disease mechanisms, identify vulnerable patients, and guide therapeutic decisions. The study by Suciu et al. exemplifies this evolution by demonstrating how natriuretic peptides and soluble ST2 improve long-term prognostic assessment beyond conventional clinical and imaging parameters (contribution 7). Likewise, advances in molecular imaging, artificial intelligence-assisted phenotyping, and biomarker discovery discussed throughout the collection suggest that future cardiovascular risk assessment will increasingly rely on multidimensional biological profiling rather than isolated measurements. This precision-oriented direction is also supported by large-scale proteomic and network-medicine studies, which demonstrate that circulating protein signatures can improve disease prediction, prioritize causal mediators, and connect molecular pathways to clinically recognizable cardiovascular phenotypes [7,8]. Spatial multi-omics adds a further layer by preserving tissue context, allowing inflammatory, metabolic, stromal, and vascular programs to be interpreted within the microenvironments in which disease actually develops [9].

Viewed collectively, this research emphasizes a broader transformation occurring throughout the field of cardiovascular medicine. Traditional boundaries separating inflammation, metabolism, fibrosis, vascular biology, and clinical cardiology are progressively disappearing, as these processes are more and more understood as components of interconnected biological networks that determine disease susceptibility, progression, and response to therapy rather than individual abnormalities.

This shift has important implications. The future challenge is no longer simply to identify additional molecular pathways but to understand how these pathways interact and how their integration can be translated into clinically meaningful strategies. Achieving this goal will require continued collaboration between basic scientists, translational researchers, clinicians, bioengineers, computational scientists, and experts in artificial intelligence. The studies presented in this Special Issue provide compelling examples of this multidisciplinary approach and illustrate the remarkable progress being made toward a more comprehensive understanding of cardiovascular disease.

As Guest Editor, I hope that the contributions collected herein not only advance current knowledge but also stimulate new questions. If there is a common message that emerges from this Special Issue, it is that cardiovascular disease is best understood not as a disorder of isolated organs, cells, or molecules, but as a consequence of complex biological interactions occurring across multiple levels of organization. Understanding—and ultimately targeting—these interactions may represent the next frontier of cardiovascular medicine.
